# Supplementary material for: Selective RNA pseudouridinylation in situ by circular gRNAs in designer organelles
Source: Nat Commun. 2024 Oct 24;15:9177. doi: 10.1038/s41467-024-53403-1 (PMC11502668; doi:10.1038/s41467-024-53403-1)
Supplement: Supplementary file 1 — Supplementary Information [file 41467_2024_53403_MOESM1_ESM.pdf]

Supplementary Material

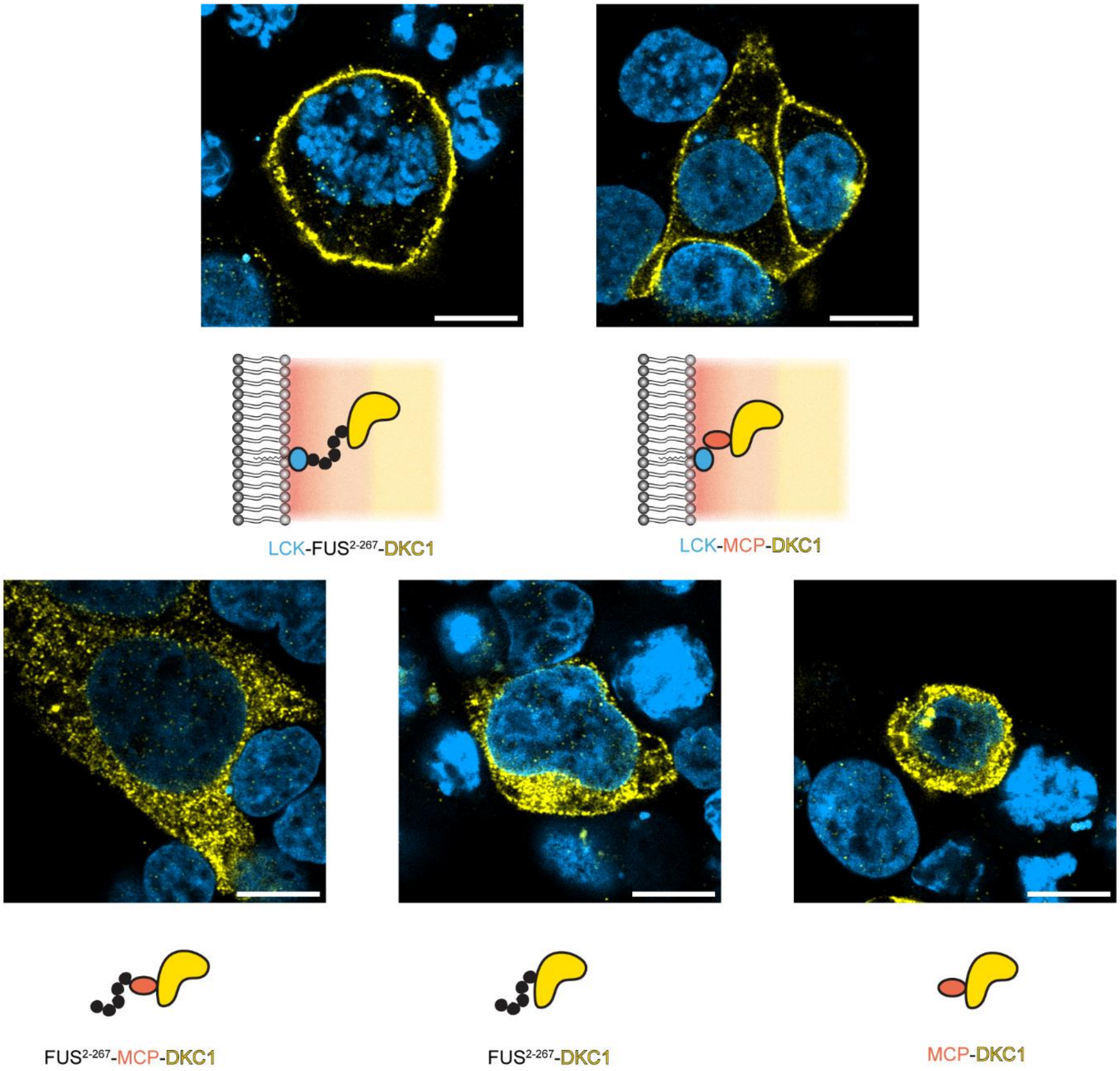

Supplementary Figure. 1. Images of fusion constructs. Immunofluorescent images of HEK293T cells transfected with constructs used in Figure 1b. Indicated fusion constructs (yellow) nuclei (blue) are shown, scale bar = 10μm.

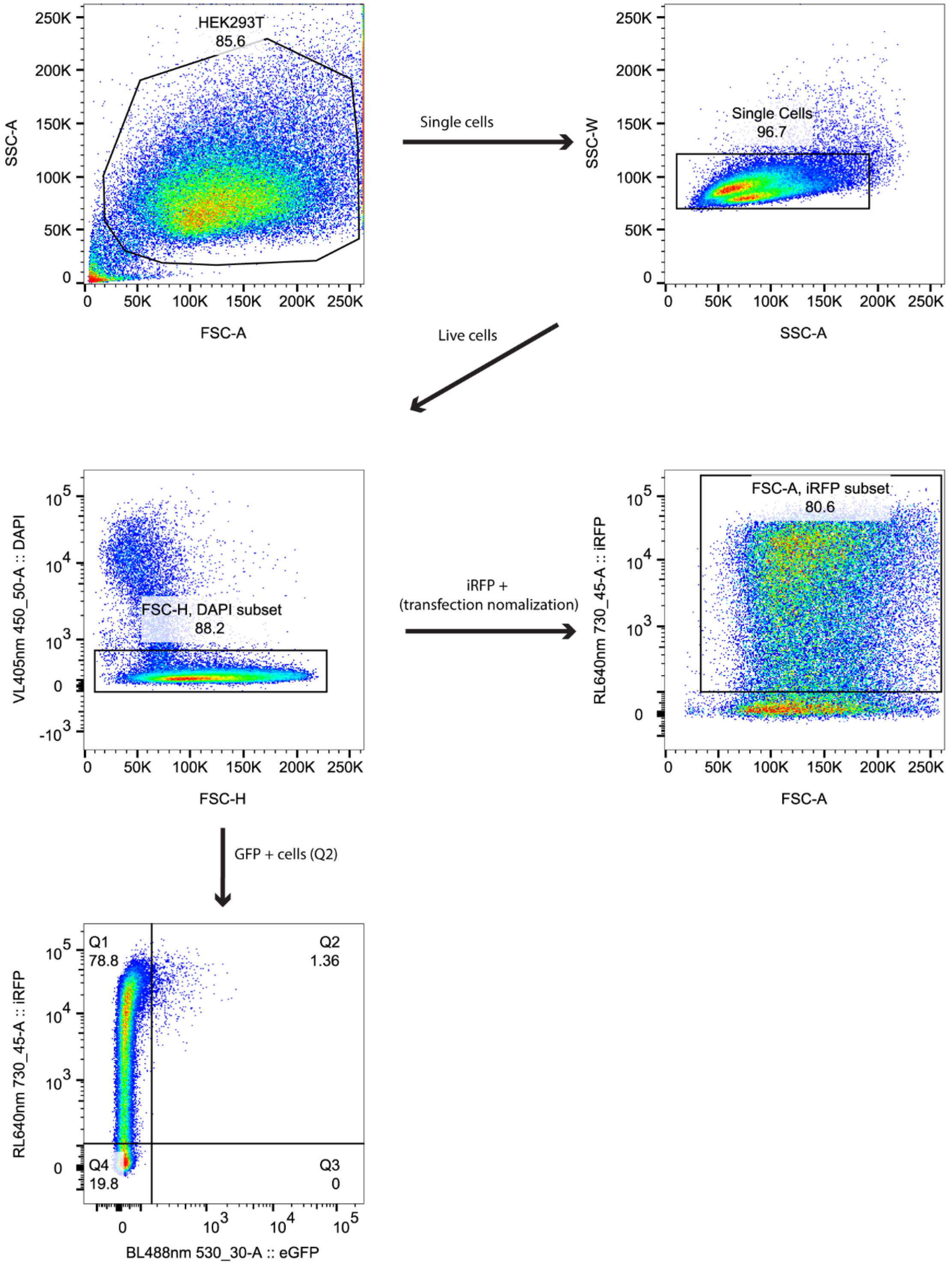

Supplementary Figure. 2. Gating strategy for flow cytometry. Representative example of a gating strategy used in flow cytometry analysis, in which HEK293T cells were transfected with the efficiency reporter only (Fig. 1b). Cells were first gated based on forward and side scatter areas to distinguish HEK293T cells (top left). Subsequently, cells were gated based on side scatter area and width to distinguish single cells (top right). Cells were then gated based on DAPI fluorescence to separate living from dead cells (middle left). From those cells, transfection efficiency was estimated by gating iRFP + cells

(middle right). Simultaneously, live cells were plotted by iRFP vs eGFP (bottom); Q1 represents iRFP cells, Q2 represents eGFP-positive cells, and Q4 represents untransfected cells.

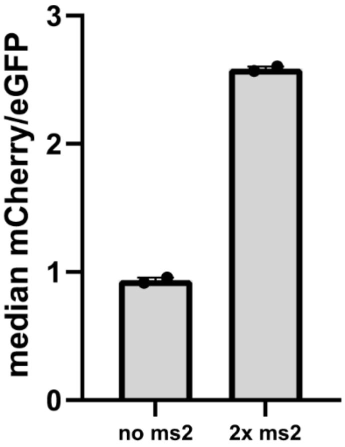

Supplementary Figure. 3. Organelle selectivity is dependent on ms2 aptamer loops. Selectivity reporter without ms2 loops abolish organelle-specific pseudouridinylation in HEK293T transfected with LCK-FUS$^{2-267}$-MCP-DKC1 and gRNAs for mCherry/eGFP. Two measurements were performed, and the SEM was plotted.

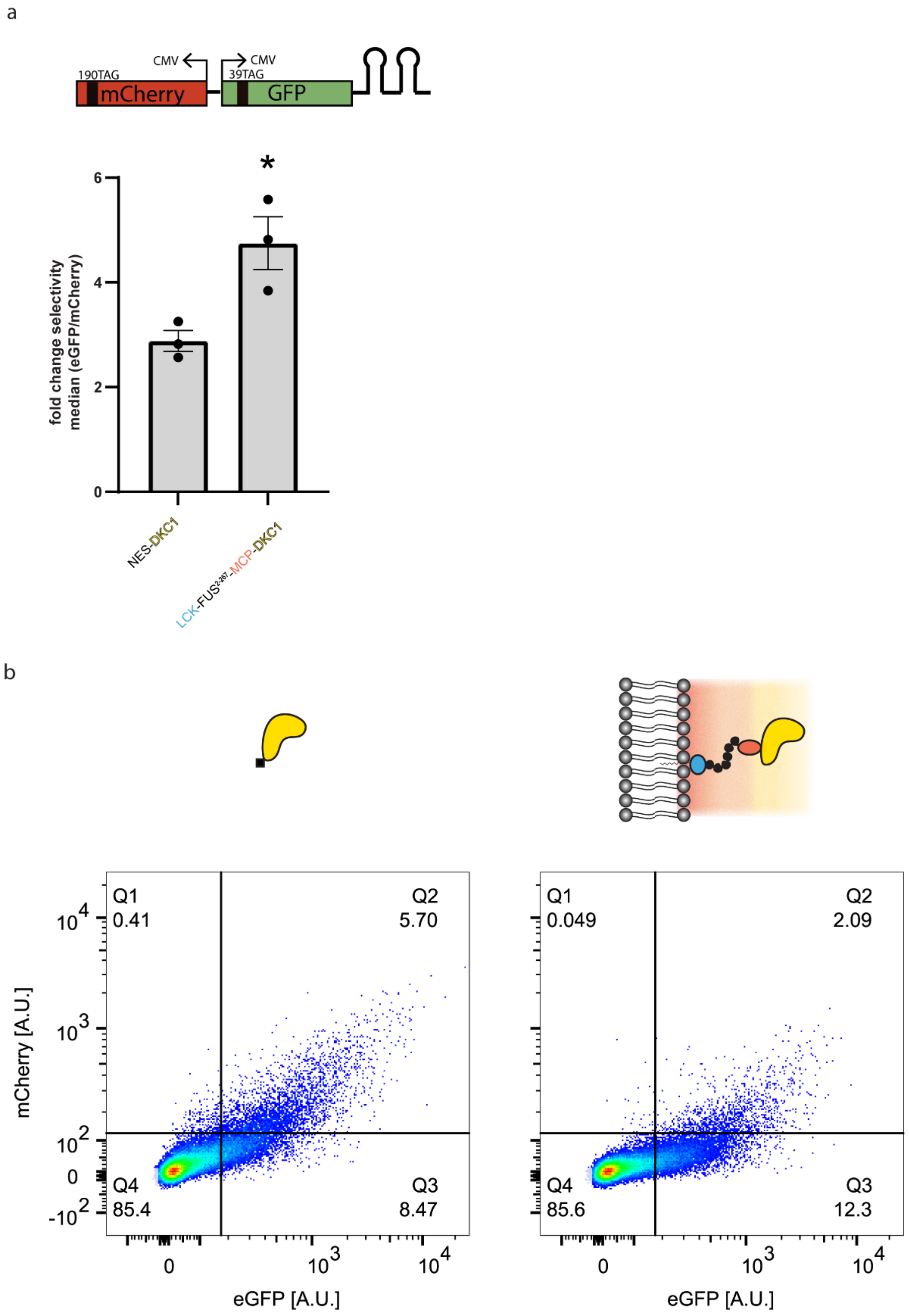

Supplementary Figure. 4. Inversion of the selectivity reporter. Pseudouridine-mediated stop-codon suppression was measured using an inverted selectivity reporter in which GFP is actively recruited via two ms2 loops. The relative fold of median mCherry/eGFP were calculated from the flow cytometry data in **b** and plotted in **a**. SEMs of three independent measurements are shown. An unpaired two-sided t-test was performed (n=3, p= 0.0263 *p<0.05).

a

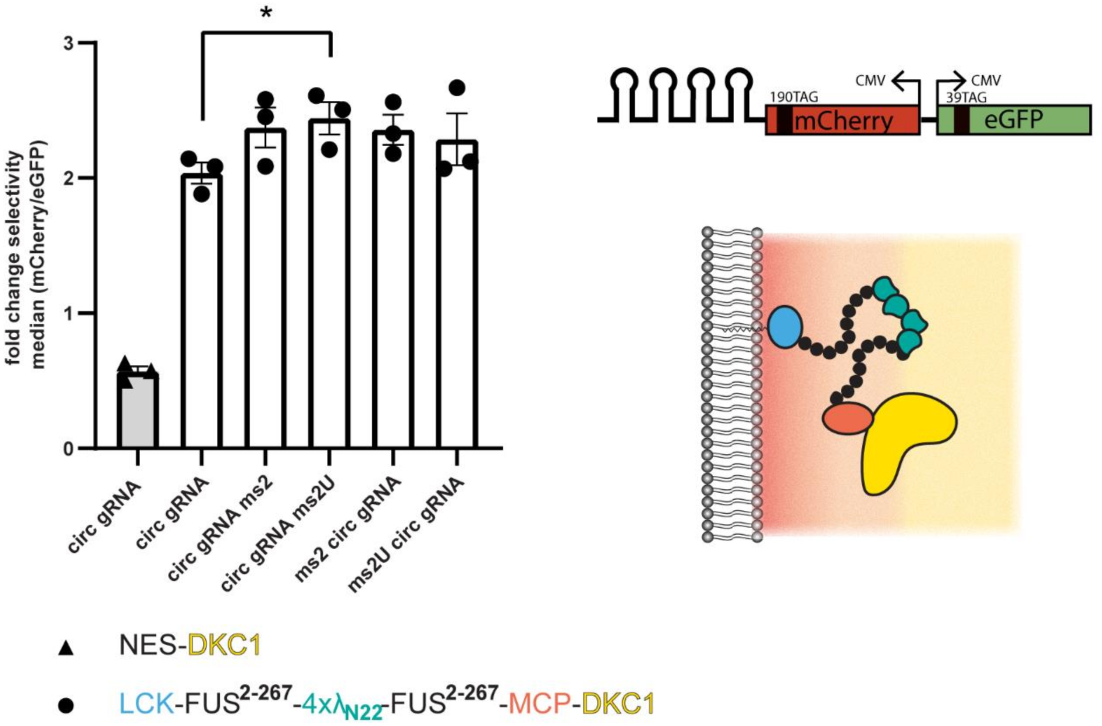

b

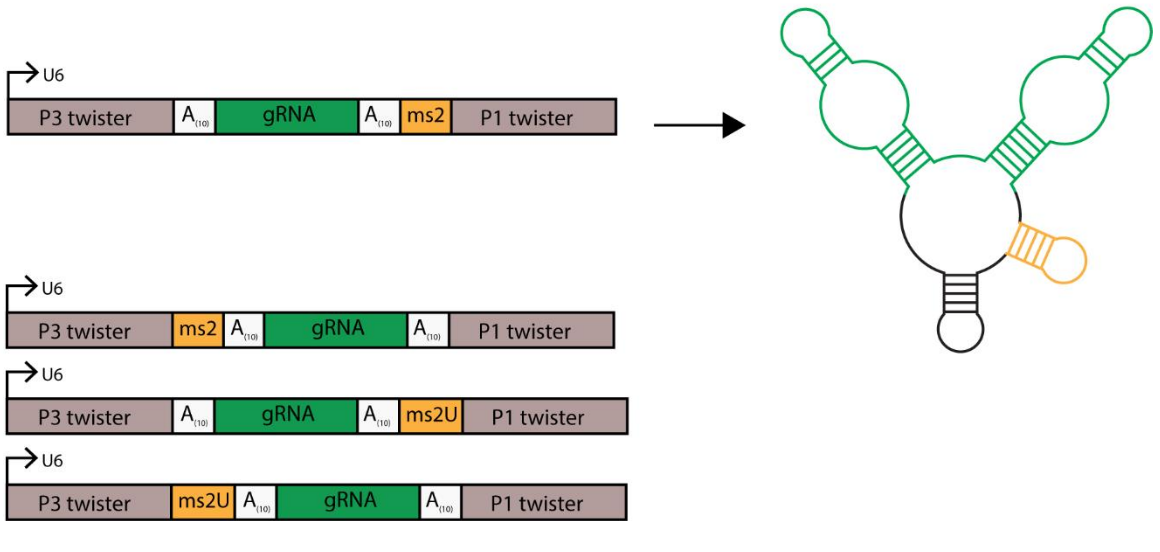

Supplementary Figure. 5. Circular gRNA tagged with ms2 loop enhances selectivity. HEK293T cells were transfected with a modified selectivity reporter in which mCherry has been tagged with four boxB loops, NES-DKC1 or LCK-FUS$^{2–267}$-4xλ_{N22}- FUS$^{2–267}$-MCP-DKC1 (shown as cartoon), and circular gRNAs for eGFP^{39TAG} and mCherry^{190TAG_4xboxB}. The circular gRNAs of mCherry include versions of ms2 loops 5' or 3' of

the gRNA sequence, as shown in **b**. The relative fold change in selectivity normalized to NES-DKC1 was plotted, an unpaired two-sided t-test was performed (n=3, p= 0.0471 *p<0.05).

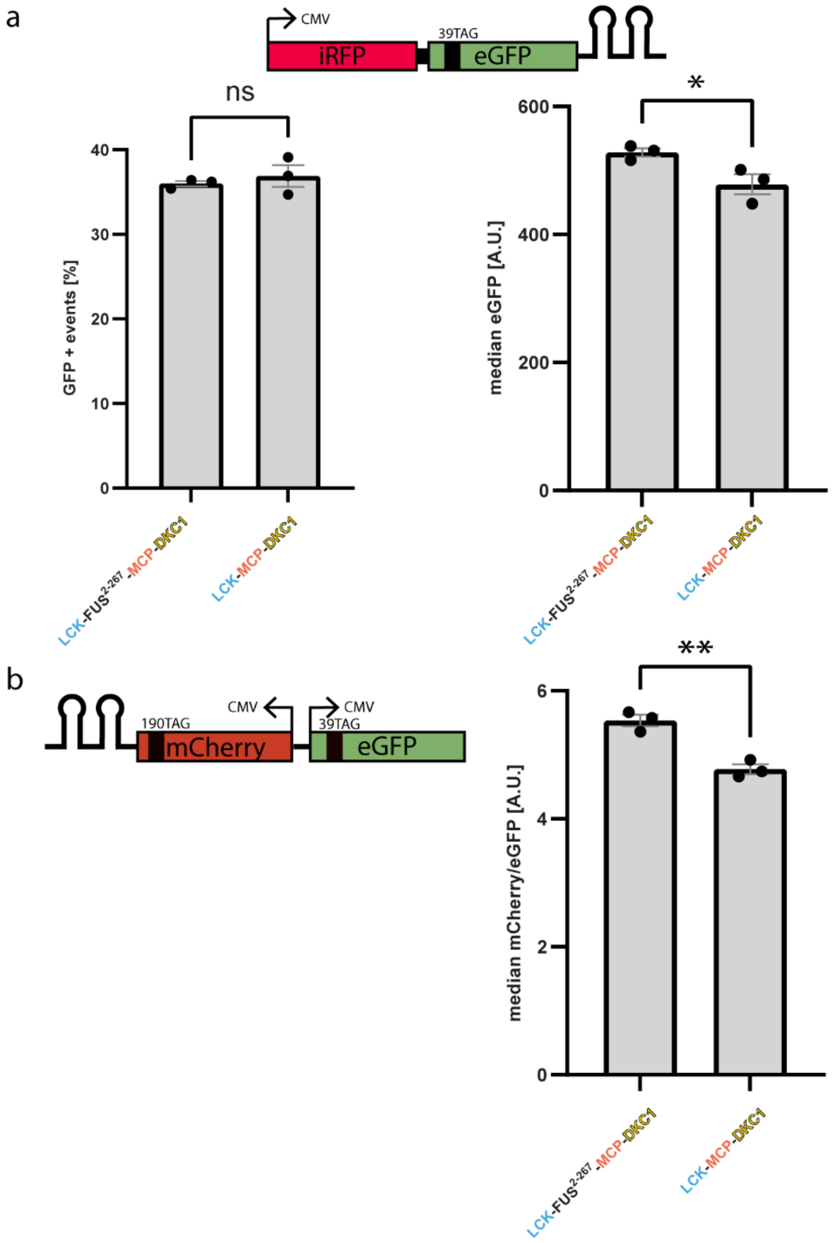

Supplementary Figure. 6. FUS$^{2-267}$ enhances organelle efficiency and specificity. (a) LCK constructs with and without FUS IDR have been transfected with the efficiency reporter aswell as circular gRNA. GFP+ events and median GFP intensities are plotted. An unpaired two-sided t-test was performed (n=3; p=0.0427, *p<0.05) and SEM was plotted, ns= non-significant (p= 0,5288). **(b)** The same contructs as in (a) have been analyzed with the selectivity reporter and circular gRNAs. Plotted is the median mCherry/eGFP ratio**.** An unpaired two-sided t-test was performed (n=3; p= 0.0031, **p<0.01) and SEM was plotted, ns= non-significant.

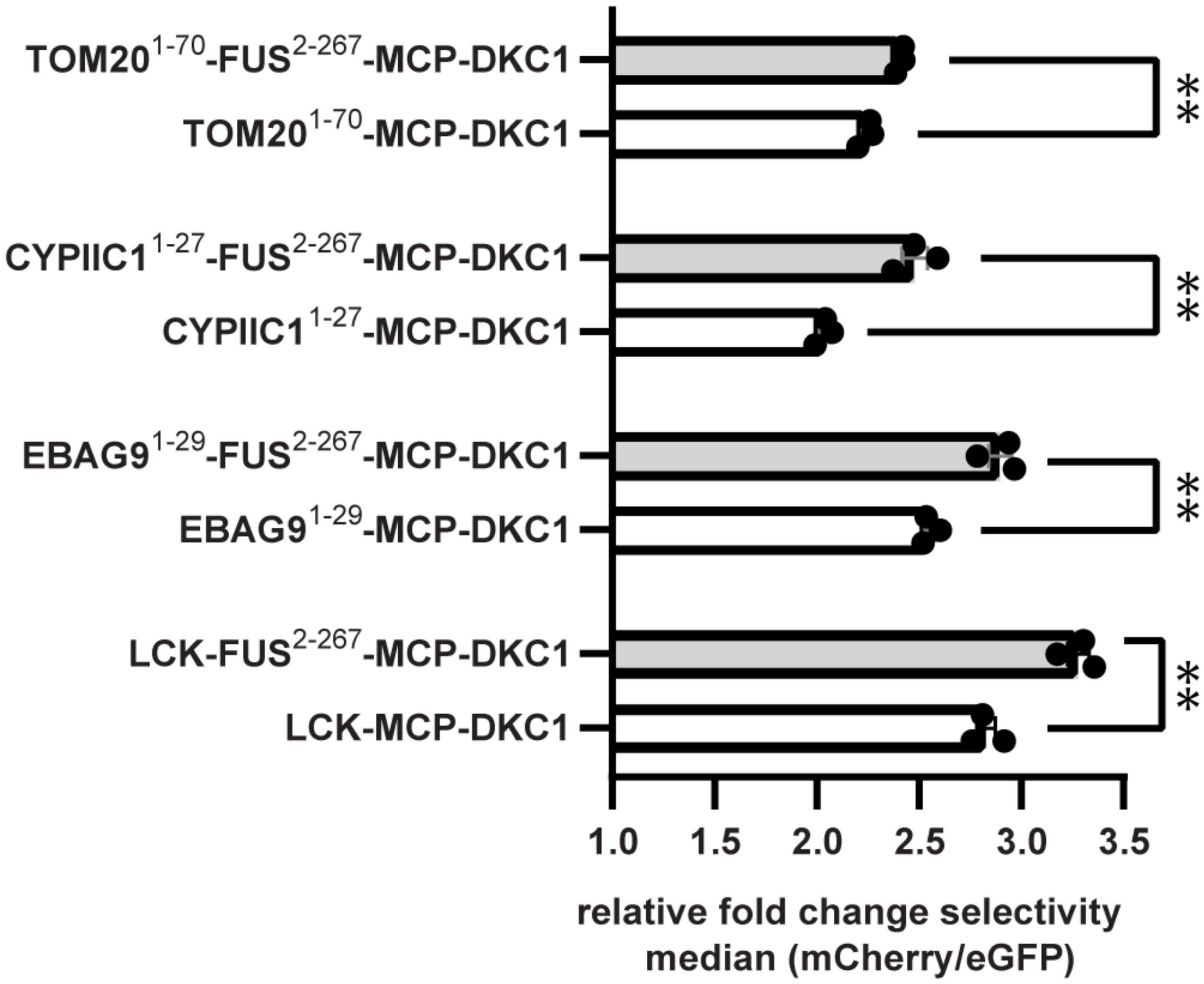

Supplementary Figure. 7. Selectivity in other membrane anchored constructs. Various N-terminal membrane anchors were tested for their selectivity using our selectivity reporter and circular gRNAs. Tom20$^{1-70}$ anchors the FUS$^{2-267}$ -MCP-DKC1 construct to mitochondria while CYPIIC1$^{1-27}$ and EBAG9$^{1-29}$ anchor them to the endoplasmic reticulum and Golgi respectively. Constructs were compared to NES-DKC1 (relative fold change of 1) and an unpaired two-sided t-test was performed (n=3, $^{**}p<0.01$). p-values are provided with Source Data file.

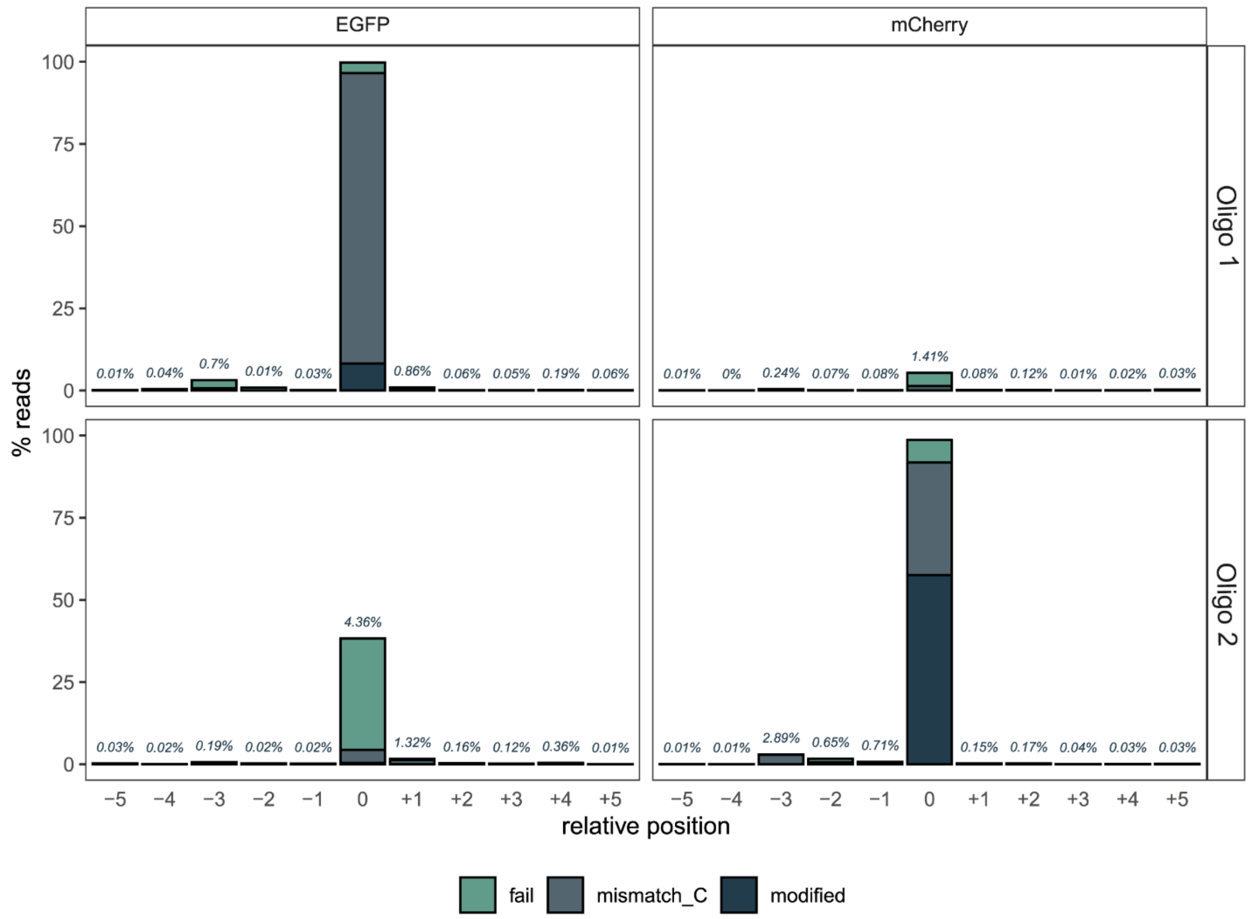

Supplementary Figure. 8. Sequence context of nanopore read covering the target region within our synthetic RNA oligos. Fully modified and non-modified RNA oligos were analyzed using DRS, U-C mismatch and modified reads were plotted. Fail refers to reads not passing the set quality threshold (see methods).

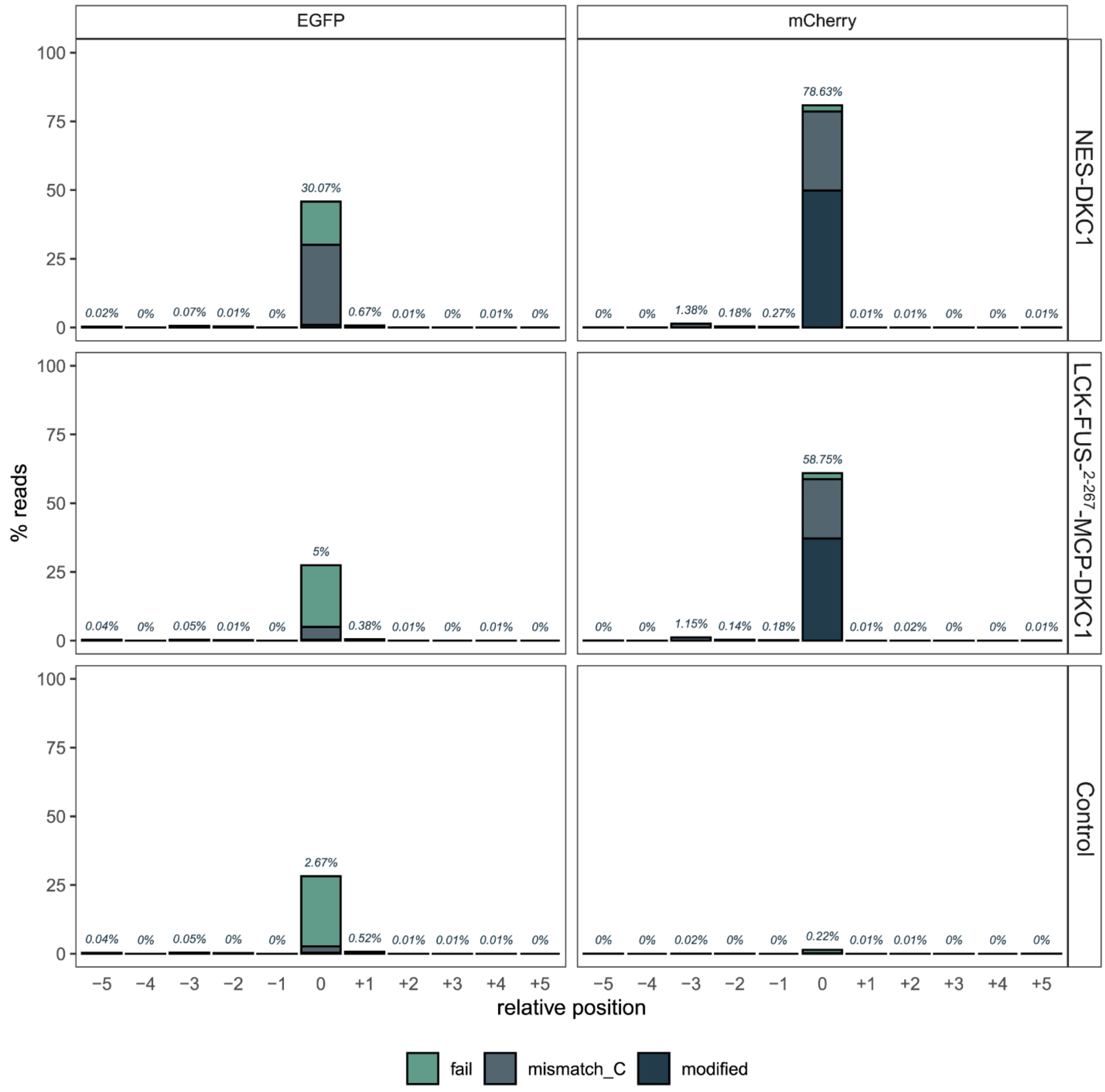

Supplementary Figure. 9. Targeted region and sequence context for sequencing experiments shown in maintext Figure 3d. The basecall accuracies for the reference sequence (−5 to +5 around the target uridine) are shown. Control refers to the use of a non-targeting guide RNA which served as a reference for the basecall error at the specific sequences. We note that absolute values cannot be compared to flow cytometry data, where photomultiplier voltages were adjusted to yield a diagonal line of fluorescence reporters to maximize dynamic range of the detection methods and to account for any differences in GFP and mCherry brightness. We also note that the mCherry target sequences shows little background in the control sequencing, while GFP shows already an elevated specific basecalling error pattern due to inherent method limitations. The values shown in the barplot in maintext Figure 4d were created by subtracting mismatch_C of the control samples. Fail refers to reads not passing the quality threshold set.

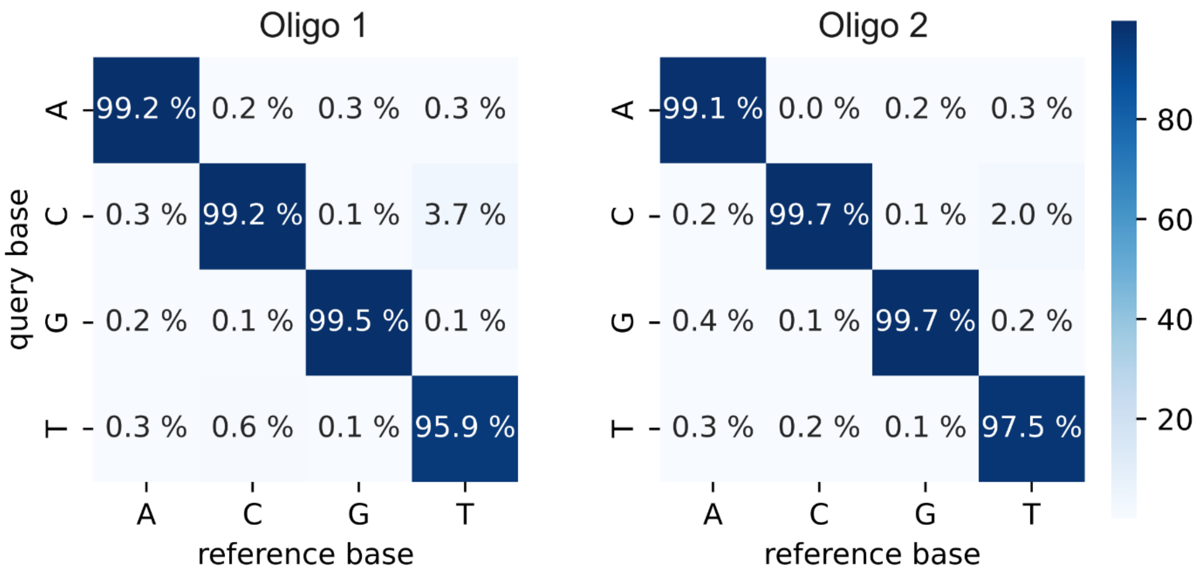

Supplementary Figure. 10. Basecall accuracy of direct RNA sequencing runs for the indicated samples (oligo 1/mCherry, oligo 2/eGFP) *x*-axis shows called bases from nanopore reads while the *y*-axis shows the reference bases of the synthetic oligos

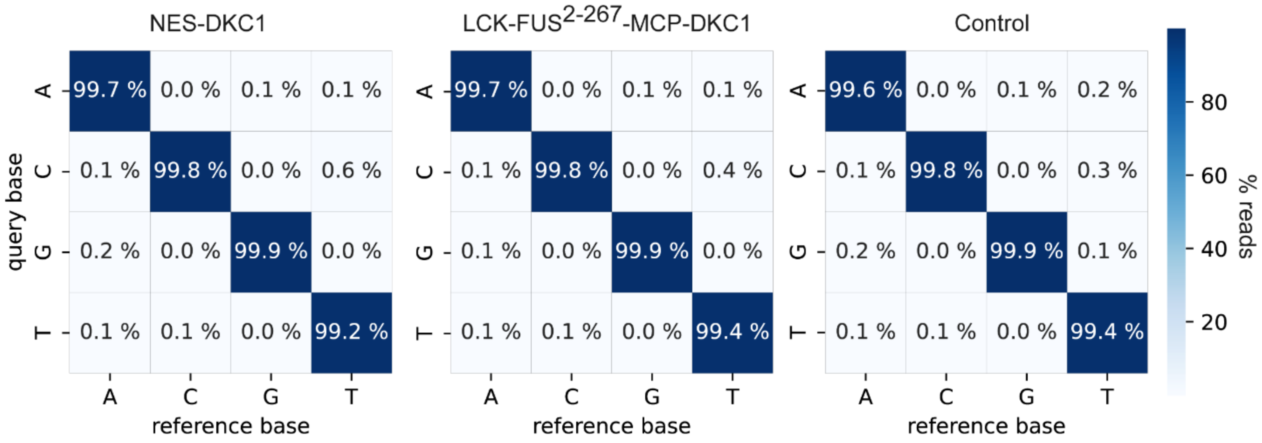

Supplementary Figure. 11. Basecall accuracy of direct RNA sequencing runs for the indicated samples, *x*-axis shows called bases from nanopore reads, while the *y*-axis shows the reference bases of the reporter. Control refers to a non-targeting gRNA.

Supplementary Table 1: DKC1 constructs

NES_DKC1(22–424)	MAYPYDVPDYAACPVPLQLPPLERLTLDLPEEDVAEIQH<br>AEEFLIKPESKVAKLDTSQWPLLLKNFDKLNVRTTHYTP<br>LACGSNPLKREIGDYIRTGFINLDKPSNPSSHEVVAWIR<br>RILRVEKTGHSGTLDPKVTGCLIVCIERATRLVKSQQSAG<br>KEYVGIVRLHNAIEGGTQLSRALETLTGALFQRPPLIAAV<br>KRQLRVRTIYESKMIEYDPERRLGIFWVSCEAGTYIRTLC<br>VHLGLLLGVGGQMQELRRVRSGVMSEKDHMVTMH<br>DVLDAQWLYDNHKDESYLRRVVYPLEKLLTSHKRLVMK<br>DSAVNAICYGAKIMLPGVLRYEDGIEVNQEIVVITTKGE<br>AICMAIALMTTAVISTCDHGIVAKIKRVIMERDTYPRK<br>WGLGPKASQKKLMIKQGLLDKHGKPTDSTPATWKQEY<br>VDYSESAKKEVVAEVVKAPQVVAEAAKTA*
LCK_FUS(2-267)_MCP_DKC1(22–424)	MGCVCSSNPEGTELASNDYTQQATQSYGAYPTQPGQ<br>GYSQQSSQPYGQQSYSGYSQSTDTSGYGQSSYSSYGQ<br>SQNTGYGTQSTPQGYGSTGGYGSSQSSQSSYGQQSSY<br>PGYGQQPAPSSTSGSYGSSSQSSSYGQPQSGSYSQQPS<br>YGGQQQSYGQQQSYNPPQGYGQQNQYNSSSGGGG<br>GGGGGGNYGQDQSSMSSGGGSGGGYGNQDQSGG<br>GGSGGYGQQDRGGRGRGGSGGGGGGGGGGYNRSS<br>GGYEPRGRGGGRGGRGGMGGSDRGGFNKFGGAIAY<br>PYDVPDYAGAPGSAGSAAGSGASNFTQFVLVDNGGT<br>GDVTVAPSNFANGIAEWISSNSRSQAYKVTCSVRQSSA<br>QNRKYTIKVEVPKGAWRSYLNMELTIPIFATNSDCELIV<br>KAMQGLLKDGNPIPSAIAANSGIYGADYKDDDDKGAP<br>GSAGSAAGSGACPVPLQLPPLERLTLDLPEEDVAEIQH<br>AEEFLIKPESKVAKLDTSQWPLLLKNFDKLNVRTTHYTP<br>LACGSNPLKREIGDYIRTGFINLDKPSNPSSHEVVAWIR<br>RILRVEKTGHSGTLDPKVTGCLIVCIERATRLVKSQQSAG<br>KEYVGIVRLHNAIEGGTQLSRALETLTGALFQRPPLIAAV<br>KRQLRVRTIYESKMIEYDPERRLGIFWVSCEAGTYIRTLC<br>VHLGLLLGVGGQMQELRRVRSGVMSEKDHMVTMH<br>DVLDAQWLYDNHKDESYLRRVVYPLEKLLTSHKRLVMK<br>DSAVNAICYGAKIMLPGVLRYEDGIEVNQEIVVITTKGE<br>AICMAIALMTTAVISTCDHGIVAKIKRVIMERDTYPRK<br>WGLGPKASQKKLMIKQGLLDKHGKPTDSTPATWKQEY<br>VDYSESAKKEVVAEVVKAPQVVAEAAKTA*
LCK_MCP_DKC1(22–424)	MGCVCSSNPEGTELASNFTQFVLVDNGGTGDVTVAPS<br>NFANGIAEWISSNSRSQAYKVTCSVRQSSAQNRKYTIK<br>VEVPKGAWRSYLNMELTIPIFATNSDCELIVKAMQGLL<br>KDGNPIPSAIAANSGIYGADYKDDDDKGAPGSAGSAA<br>GSGACPVPLQLPPLERLTLDLPEEDVAEIQHAEEFLIKPE<br>SKVAKLDTSQWPLLLKNFDKLNVRTTHYTPLACGSNPL<br>KREIGDYIRTGFINLDKPSNPSSHEVVAWIRRILRVEKTG<br>HSGTLDPKVTGCLIVCIERATRLVKSQQSAGKEYVGIVR<br>LHNAIEGGTQLSRALETLTGALFQRPPLIAAVKRQLRVR<br>TIYESKMIEYDPERRLGIFWVSCEAGTYIRTLCVHLGLLL

	GVGGQMQELRRVRSGVMSEKDHMVTMHDVLDAQ WLYDNHKDESYLRRVVYPLEKLLTSHKRLVMKDSAVNA ICYGAKIMLPGVLRYEDGIEVNQEIVVITTKGEAICMAIA LMTTAVISTCDHGIVAKIKRVIMERDTYPRKWGLGPKA SQKKLMIKQGLLDKHGKPTDSTPATWKQEYVDYSESAK KEVVAEVVKAPQVVAEAAKTA*
LCK_FUS(2-267)_DKC1(22–424)	MGCVCSSNPEGTELASNDYTQQATQSYGAYPTQPGQ GYSQQSSQPYGQQSYSGYSQSTDTSGYGQSSYSSYGQ SQNTGYGTQSTPQGYGSTGGYGSSQSSQSSYGQQSSY PGYGQQPAPSSTSGSYGSSSQSSSYGQPQSGSYSQQPS YGGQQQSYGQQQSYNPPQGYGQQNQYNSSSGGGG GGGGGGNYGQDQSSMSSGGGSGGGYGNQDQSGG GGSGGYGQQDRGGRGRGGSGGGGGGGGGGYNRSS GGYEPRGRGGGRGGRGGMGGSDRGGFNKFGGDYK DDDDKGAPGSAGSAAGSGACPVPLQLPPLERLTLDLPE EDVAEIQHAEEFLIKPESKVAKLDTSQWPLLLKNFDKLN VRTTHYTPLACGSNPLKREIGDYIRTGFINLDKPSNPSS HEVVAWIRRILRVEKTGHSGTLDPKVTGCLIVCIERATRL VKSQQSAGKEYVGIVRLHNAIEGGTQLSRALETLTGALF QRPPLIAAVKRQLRVRTIYESKMIEYDPERRLGIFWVSC EAGTYIRTLCVHLGLLLGVGGQMQELRRVRSGVMSEK DHMVTMHDVLDAQWLYDNHKDESYLRRVVYPLEKLL TSHKRLVMKDSAVNAICYGAKIMLPGVLRYEDGIEVNQ EIVVITTKGEAICMAIALMTTAVISTCDHGIVAKIKRVIM ERDTYPRKWGLGPKASQKKLMIKQGLLDKHGKPTDST PATWKQEYVDYSESAKKEVVAEVVKAPQVVAEAAKTA *
FUS(2-267)_MCP_DKC1(22–424)	MASNDYTQQATQSYGAYPTQPGQGYSQQSSQPYGQ QSYSGYSQSTDTSGYGQSSYSSYGQSQNTGYGTQSTP QGYGSTGGYGSSQSSQSSYGQQSSYPGYGQQPAPSST SGSYGSSSQSSSYGQPQSGSYSQQPSYGGQQQSYGQ QQSYNPPQGYGQQNQYNSSSGGGGGGGGGGNYGQ DQSSMSSGGGSGGGYGNQDQSGGGGSGGYGQQDR GGRGRGGSGGGGGGGGGGYNRSSGGYEPRGRGGG RGGRGGMGGSDRGGFNKFGGAIAYPYDVPDYAGAPG SAGSAAGSGASNFTQFVLVDNGGTGDVTVAPSNFAN GIAEWISSNSRSQAYKVTCSVRQSSAQNRKYTIKVEVPK GAWRSYLNMELTIPIFATNSDCELIVKAMQGLLKDGNP IPSAIAANSGIYGADYKDDDDKGAPGSAGSAAGSGACP VPLQLPPLERLTLDLPEEDVAEIQHAEEFLIKPESKVAKL DTSQWPLLLKNFDKLNVRTTHYTPLACGSNPLKREIGD YIRTGFINLDKPSNPSSHEVVAWIRRILRVEKTGHSGTL DPKVTGCLIVCIERATRLVKSQQSAGKEYVGIVRLHNAIE GGTQLSRALETLTGALFQRPPLIAAVKRQLRVRTIYESK MIEYDPERRLGIFWVSCEAGTYIRTLCVHLGLLLGVGG QMQELRRVRSGVMSEKDHMVTMHDVLDAQWLYDN HKDESYLRRVVYPLEKLLTSHKRLVMKDSAVNAICYGAK IMLPGVLRYEDGIEVNQEIVVITTKGEAICMAIALMTTA VISTCDHGIVAKIKRVIMERDTYPRKWGLGPKASQKKL

	MIKQGLLDKHGKPTDSTPATWKQEYVDYSESAKKEVVAEVVKAPQVVAEAAKTA*
FUS(2-267)_DKC1(22–424)	MASNDYTQQATQSYGAYPTQPGQGYSQQSSQPYGQQSYSGYSQSTDTSGYGQSSYSSYGQSQNTGYGTQSTPQGYGSTGGYGSSQSSQSSYGQQSSYPGYGQQPAPSSTSGSYGSSSQSSSYGQPQSGSYSQQPSYGGQQQSYGQQQSYNPPQGYGQQNQYNSSSGGGGGGGGGGNYGQDQSSMSSGGGSGGGYGNQDQSGGGGSGGYGQQDRGGRGRGGSGGGGGGGGGGYNRSSGGYEPRGRGGGRGGRGGMGGSDRGGFNKFGGDYKDDDDKGAPGSAGSAAGSGACPVPLQLPPLERLTLDLPEEDVAEIQHAEEFLIKPESKVAKLDTSQWPLLLKNFDKLNVRTTHYTPLACGSNPLKREIGDYIRTGFINLDKPSNPSSHEVVAWIRRILRVEKTGHSGTLDPKVTGCLIVCIERATRLVKSQQSAGKEYVGIVRLHNAIEGGTQLSRALETLTGALFQRPPLIAAVKRQLRVRTIYESKMIEYDPERRLGIFWVSCEAGTYIRTLCVHLGLLLGVGGQMQELRRVRSGVMSEKDHMVTMHDVLDAQWLYDNHKDESYLRRVVYPLEKLLTSHKRLVMKDSAVNAICYGAKIMLPGVLRYEDGIEVNQEIVVITTKGEAICMAIALMTTAVISTCDHGIVAKIKRVIMERDTYPRKWGLGPKASQKKLMIKQGLLDKHGKPTDSTPATWKQEYVDYSESAKKEVVAEVVKAPQVVAEAAKTA*
MCP_DKC1(22–424)	MASNFTQFVLVDNGGTGDVTVAPSNFANGIAEWISSNSRSQAYKVTCSVRQSSAQNRKYTIKVEVPKGAWRSYLNMELTIPIFATNSDCELIVKAMQGLLKDGNPIPSAIAANSGIYGADYKDDDDKGAPGSAGSAAGSGACPVPLQLPPLERLTLDLPEEDVAEIQHAEEFLIKPESKVAKLDTSQWPLLLKNFDKLNVRTTHYTPLACGSNPLKREIGDYIRTGFINLDKPSNPSSHEVVAWIRRILRVEKTGHSGTLDPKVTGCLIVCIERATRLVKSQQSAGKEYVGIVRLHNAIEGGTQLSRALETLTGALFQRPPLIAAVKRQLRVRTIYESKMIEYDPERRLGIFWVSCEAGTYIRTLCVHLGLLLGVGGQMQELRRVRSGVMSEKDHMVTMHDVLDAQWLYDNHKDESYLRRVVYPLEKLLTSHKRLVMKDSAVNAICYGAKIMLPGVLRYEDGIEVNQEIVVITTKGEAICMAIALMTTAVISTCDHGIVAKIKRVIMERDTYPRKWGLGPKASQKKLMIKQGLLDKHGKPTDSTPATWKQEYVDYSESAKKEVVAEVVKAPQVVAEAAKTA*
LCK_FUS(2-267)_4xλN22_FUS(2-267)_MCP_DKC1(22–424)	MGCVCSSNPEGTELASNDYTQQATQSYGAYPTQPGQGYSQQSSQPYGQQSYSGYSQSTDTSGYGQSSYSSYGQSQNTGYGTQSTPQGYGSTGGYGSSQSSQSSYGQQSSYPGYGQQPAPSSTSGSYGSSSQSSSYGQPQSGSYSQQPSYGGQQQSYGQQQSYNPPQGYGQQNQYNSSSGGGGGGGGGGNYGQDQSSMSSGGGSGGGYGNQDQSGGGGSGGYGQQDRGGRGRGGSGGGGGGGGGGYNRSSGGYEPRGRGGGRGGRGGMGGSDRGGFNKFGGLATMDAQTRRRERRAEKQAQWKAANPPLDGAGAGAGAGAGAGGLATMDAQTRRRERRAEKQAQWKAANPPLDGAGAGAGAGAGAGGLATMDAQTRRRERRAEKQAQ

	WKAANPPLDGAGAGAGAGAGAGGLATMDAQTRRR<br>ERRAEKQAQWKAANPPLEAIAASNDYTQQATQSYGAY<br>PTQPGQGYSQQSSQPYGQQSYSGYSQSTDTSGYGQSS<br>YSSYGQSQNTGYGTQSTPQGYGSTGGYGSSQSSQSSY<br>GQQSSYPGYGQQPAPSSTSGSYGSSSQSSSYGQPQSG<br>SYSQQPSYGGQQQSYGQQQSYNPPQGYGQQNQYNS<br>SSGGGGGGGGGGNYGQDQSSMSSGGGSGGGYGNQ<br>DQSGGGGSGGYGQQDRGGRGRGGSGGGGGGGGG<br>GYNRSSGGYEPRGRGGGRGGRGGMGGSDRGGFNKF<br>GGASNFTQFVLVDNGGTGDVTVAPSNFANGIAEWISS<br>NSRSQAYKVTCSVRQSSAQNRKYTIKVEVPKGAWRSYL<br>NMELTIPIFATNSDCELIVKAMQGLLKDGNPIPSAIAAN<br>SGIYGADYKDDDDKGAPGSAGSAAGSGACPVPLQLPP<br>LERLTLDLPEEDVAEIQHAEEFLIKPESKVAKLDTSQWPL<br>LLKNFDKLNVRTTHYTPLACGSNPLKREIGDYIRTGFINL<br>DKPSNPSSHEVVAWIRRILRVEKTGHSGTLDPKVTGCLI<br>VCIERATRLVKSQQSAGKEYVGIVRLHNAIEGGTQLSRA<br>LETLTGALFQRPPLIAAVKRQLRVRTIYESKMIEYDPERR<br>LGIFWVSCEAGTYIRTLCVHLGLLLGVGGQMQELRRVR<br>SGVMSEKDHMVTMHDVLDAQWLYDNHKDESYLRRV<br>VYPLEKLLTSHKRLVMKDSAVNAICYGAKIMLPGVLRYE<br>DGIEVNQEIVVITTKGEAICMAIALMTTAVISTCDHGIV<br>AKIKRVIMERDTYPRKWGLGPKASQKKLMIKQGLLDK<br>HGKPTDSTPATWKQEYVDYSESAKKEVVAEVVKAPQV<br>VAEAAKTA*

Supplementary Table 2: reporter

**iRFP_eGFP(39TAG)_2xms2**	acatgggtgatcctcatgttttctagagtcgacctgcagacatgggtg<br>atcctcatgttttctaggcaattaggtaccttaggatccttagtgatggt<br>gatggtgatgcttgtacagctcgtccatgccgagagtgatcccggcgg<br>cggtcacgaactccagcaggaccatgtgatcgcgcttctcgttggggt<br>ctttgctcagggcggactgggtgctcaggtagtggttgtcgggcagca<br>gcacggggccgtcgccgatgggggtgttctgctggtagtggtcggcg<br>agctgcacgctgccgtcctcgatgttgtggcggatcttgaagttggcct<br>tgatgccgttcttctgcttgtcggccatgatatagacgttgtggctgttg<br>tagttgtactccagcttgtgccccaggatgttgccgtcctccttgaagtc<br>gatgcccttcagctcgatgcggttcaccagggtgtcgccctcgaacttc<br>acctcggcgcgggtcttgtagttgccgtcgtccttgaagaagatggtg<br>cgctcctggacgtagccttcgggcatggcggacttgaagaagtcgtg<br>ctgcttcatgtggtcggggtagcggctgaagcactgcacgccgtagg<br>tcagggtggtcacgagggtgggccagggcacgggcagcttgccggt<br>ggtgcagatgaacttcagggtcagcttgccctaggtggcatcgccctc<br>gccctcgccggacacgctgaacttgtggccgtttacgtcgccgtccag<br>ctcgaccaggatgggcaccaccccggtgaacagctcctcgcccttgc<br>tcactttatcatcatcatctttgtaatcggtaccggccgccgctttggcc<br>gccgcttctttggccgccgcttctttggccgccgcttctttggccgccgc<br>ttccgccagctcttccatcacgccgatctgccaggccagaacctgggc

	gactagctcgcaggcttggcggccatcgagatcgacgtagtacggcg<br>ttcggtgatggcaaacgatcaatccccacagtcgctcgccgcgcaaa<br>atcgagatcgacatcgtgccgtgcatgcctatgttgcgcatgaattcc<br>agatggacgggcgagacgctgcgcaggatggcgaagctaagatca<br>atcggccgcccggtgaccggattgaggtctggggtgaccggcaccg<br>gccgataattgatatcgggaatgatccgtaccgggttgatggtataga<br>gccgacgggcctgcgccggcacggttgaggcaggatagtgcaggcc<br>tagttttgactcgacctcggcgcaccgatcctctgcgatcacttcgccg<br>ctgaagtcggaggcgaagcgatagatcatcacccgatcgaagccgg<br>taatcttccgcacctcttgcgccgcggcggcgcaggcgctttccaagg<br>tttcggcggcctgcaggcggcggatggcgctgttggtgcggcggaag<br>aacgcctgcggctcggcgacgtcccgctggggaggctcgagctcga<br>ggaagatgagctgatcatggcgatgccaggagccgatgaagcctgc<br>gtcctttcgcatcgtgaagccgacagtgatcggtgctccgacggccgc<br>cccgggctcggccaaggcgatcgtcagacggttgtgcgtctccgagt<br>cgaagacatcggccgcagagcggccgatcagggcgccgatcgccag<br>tccggtgagttcgggaaggttgtcgctgccggcaacgatcgtcatgtc<br>ggcggcgagggcgagcagcagtccatgcggttggatggcaccgggg<br>atatggatcggctcatcgtcgcaggtcaagaggtcaggctgcctggc<br>gacggatccttccgcgctcgcgctatcttccactttgcgttttttttcgg<br>cggggtgctttccaggcggcccat
**eGFP(39TAG)**	atgggccgcctggaaagcaccccgccgaaaaaaaaacgcaaagtg<br>gaagatagcgcgagcgattacaaagatgatgatgataaagtgagca<br>agggcgaggagctgttcaccggggtggtgcccatcctggtcgagctg<br>gacggcgacgtaaacggccacaagttcagcgtgtccggcgagggcg<br>agggcgatgccacctagggcaagctgaccctgaagttcatctgcacc<br>accggcaagctgcccgtgccctggcccaccctcgtgaccaccctgac<br>ctacggcgtgcagtgcttcagccgctaccccgaccacatgaagcagc<br>acgacttcttcaagtccgccatgcccgaaggctacgtccaggagcgc<br>accatcttcttcaaggacgacggcaactacaagacccgcgccgaggt<br>gaagttcgagggcgacaccctggtgaaccgcatcgagctgaagggc<br>atcgacttcaaggaggacggcaacatcctggggcacaagctggagt<br>acaactacaacagccacaacgtctatatcatggccgacaagcagaa<br>gaacggcatcaaggccaacttcaagatccgccacaacatcgaggac<br>ggcagcgtgcagctcgccgaccactaccagcagaacacccccatcg<br>gcgacggccccgtgctgctgcccgacaaccactacctgagcacccag<br>tccgccctgagcaaagaccccaacgagaagcgcgatcacatggtcc<br>tgctggagttcgtgaccgccgccgggatcactctcggcatggacgag<br>ctgtacaagGAGCAGAAACTCATCTCTGAAGAGGATCT<br>GGAGCAGAAACTCATCTCTGAAGAGGATCTGtaa
**mCherry(190TAG)_2xms2**	atgggccgcctggaaagcaccccgccgaaaaaaaaacgcaaagtg<br>gaagatagcgcgagcgtgagcaagggcgaggaggataacatggcc<br>atcatcaaggagttcatgcgcttcaaggtgcacatggagggctccgt<br>gaacggccacgagttcgagatcgagggcgagggcgagggccgccc<br>ctacgagggcacccagaccgccaagctgaaggtgaccaagggtgg<br>cccctgcccttcgcctgggacatcctgtcccctcagttcatgtacggc<br>tccaaggcctacgtgaagcaccccgccgacatccccgactacttgaa<br>gctgtccttccccgagggcttcaagtgggagcgcgtgatgaacttcga<br>ggacggcggcgtggtgaccgtgacccaggactcctccctgcaggac

	ggcgagttcatctacaaggtgaagctgcgcggcaccaacttcccctc<br>cgacggccccgtaatgcagaagaagacgatgggctgggaggcctcc<br>tccgagcggatgtaccccgaggacggcgccctgaagggcgagatca<br>agcagaggctgaagctgaaggacggcggccactacgacgctgagg<br>tcaagaccacctacaaggccaagtagcccgtgcagctgcccggcgc<br>ctacaacgtcaacatcaagttggacatcacctcccacaacgaggact<br>acaccatcgtggaacagtacgaacgcgccgagggccgccactccac<br>cggcggcatggacgagctgtacaagcatcatcatcatcatcattaaa<br>gatcctaaggtacctaattgcctagaaaacatgaggatcacccatgt<br>ctgcaggtcgactctagaaaacatgaggatcacccatgt
**mCherry(190TAG)_4xboxb**	atgggccgcctggaaagcaccccgccgaaaaaaaaacgcaaagtg<br>gaagatagcgcgagcgtgagcaagggcgaggaggataacatggcc<br>atcatcaaggagttcatgcgcttcaaggtgcacatggagggctccgt<br>gaacggccacgagttcgagatcgagggcgagggcgagggccgccc<br>ctacgagggcacccagaccgccaagctgaaggtgaccaagggtgg<br>cccccctgcccttcgcctgggacatcctgtcccctcagttcatgtacggc<br>tccaaggcctacgtgaagcaccccgccgacatccccgactacttgaa<br>gctgtccttccccgagggcttcaagtgggagcgcgtgatgaacttcga<br>ggacggcggcgtggtgaccgtgacccaggactcctccctgcaggac<br>ggcgagttcatctacaaggtgaagctgcgcggcaccaacttcccctc<br>cgacggccccgtaatgcagaagaagacgatgggctgggaggcctcc<br>tccgagcggatgtaccccgaggacggcgccctgaagggcgagatca<br>agcagaggctgaagctgaaggacggcggccactacgacgctgagg<br>tcaagaccacctacaaggccaagtagcccgtgcagctgcccggcgc<br>ctacaacgtcaacatcaagttggacatcacctcccacaacgaggact<br>acaccatcgtggaacagtacgaacgcgccgagggccgccactccac<br>cggcggcatggacgagctgtacaagcatcatcatcatcatcattaaa<br>gatcctaaggtaccGCCCTGAAAAAGGGCTCGAGCCCTG<br>AAAAAGGGCAATTGCCCTGAAAAAGGGCGTCGACG<br>CCCTGAAAAAGGGC
**AldoB(148TGA)_2xms2**	ATGGCCCAcCGATTTCCAGCCCTCACCCAGGAGCAGA<br>AGAAGGAGCTCTCAGAAATTGCCCAGAGCATTGTTG<br>CCAATGGAAAGGGGATCCTGGCTGCAGATGAATCTG<br>TAGGTACCATGGGGAACCGCCTGCAGAGGATCAAGG<br>TGGAAAACACTGAAGAGAACCGCCGGCAGTTCCGA<br>GAAATCCTCTTCTCTGTGGACAGTTCCATCAACCAGA<br>GCATCGGGGGTGTGATCCTTTTCCACGAGACCCTCTA<br>CCAGAAGGACAGCCAGGGAAAGCTGTTCAGAAACA<br>TCCTCAAGGAAAAGGGGATCGTGGTGGGAATCAAGT<br>TAGACCAAGGAGGTGCTCCTCTTGCAGGAACAAACA<br>AAGAAACCACCATTCAAGGGCTTGATGGCCTCTCAG<br>AGCGCTGTGCTCAGTACAAGAAAGATGGTGTTGACT<br>TTGGGAAGTGACGTGCTGTGCTGAGGATTGCCGACC<br>AGTGTCCATCCAGCCTCCCTATCCAGGAAAACGCCAA<br>CGCCCTGGCTCGCTACGCCAGCATCTGTCAGCAGAAT<br>GGACTGGTACCTATTGTTGAACCAGAGGTAATTCCTG<br>ATGGAGACCATGACCTGGAACACTGCCAGTATGTTAC<br>TGAGAAGGTCCTGGCTGCTGTCTACAAGGCCCTGAA<br>TGACCATCATGTTTACCTGGAGGGCACCCTGCTAAAG

	CCCAACATGGTGACTGCTGGACATGCCTGCACCAAG AAGTATACTCCAGAACAAGTAGCTATGGCCACCGTAA CAGCTCTCCACCGTACTGTTCCTGCAGCTGTTCCTGG CATCTGCTTTTTGTCTGGTGGCATGAGTGAAGAGGAT GCCACCCTCAACCTCAATGCTATCAACCTTTGCCCTCT ACCAAAGCCCTGGAAACTAAGTTTCTCTGGGGTGGC AAGGCTGcaaacaaGGAGGCAACCCAGGAGGCTTTTA GAGCAGAAACTCATCTCTGAAGAGGATCTGGAGCAG AAACTCATCTCTGAAGAGGATCTGtaaagcttTGTACAc ctagaaaacatgaggatcacccatgtctgcacctcgacactagaaaa catgaggatcacccatgt

Supplementary Table 3: gRNAs

**gRNA(eGFP39TAG)**	GTGGGAGATTTGCCCTCGGACAGAGAGAAACTCTGC TGTGTCTGAGGTGGGATCTCCCTATAGTGACCCTGCC TTCTTGCCCCCGGGACGAAATGAGAGAAATCGTAAC GGGGTGGCATGAGGCAGACAGGT
**gRNA(mCherry190TAG)**	GTGGGAGATTcgggcTCGGACAGAGAGAAACTCTGCT GTGTCTGActtggGATCTCCCTATAGTGACCCTGCCTTca cgggcCCGGGACGAAATGAGAGAAATCGTAACGGcttg gcctGAGGCAGACAGGT
**gRNA(Scramble)**	GTGGGAGATTTATTGTCGGACAGAGAGAAACTCTGC TGTGTCTGAAGGCAGATCTCCCTATAGTGACCCTGCC TTgcGACTGCCGGGACGAAATGAGAGAAATCGTAAC GGATGCGGAAGAGGCAGACAGGT
**Circ_gRNA(eGFP39TAG)**	GCCATCAGTCGCCGGTCCCAAGCCCGGATAAAATGG GAGGGGGCGGGAAACCGCCTAACCATGCCGACTGAT GGCAGAAAAAAAAAAGTGGGAGATTTGCCCTCGGA CAGagagAAACTCTGCTGTGTCTGAGGTGGGATCTCC CTATAGTGACCCTGCCTTCTTGCCCCCGGGACGAAAT GAGAGAAATCGTAACGGGGTGGCATGAGGCAGACA GGTAAAAAAAAAACTGCCATCAGTCGGCGTGGACTG TAGAACACTGCCAATGCCGGTCCCAAGCCCGGATAA AAGTGGAGGGTACAGTCCACGC
**Circ_gRNA(mCherry190TAG)**	GCCATCAGTCGCCGGTCCCAAGCCCGGATAAAATGG GAGGGGGCGGGAAACCGCCTAACCATGCCGACTGAT GGCAGAAAAAAAAAAGTGGGAGATTcgggcTCGGAC AGAGAGAAACTCTGCTGTGTCTGActtggGATCTCCCT ATAGTGACCCTGCCTTcacgggcCCGGGACGAAATGAG AGAAATCGTAACGGcttggcctGAGGCAGACAGGTAAA AAAAAAACTGCCATCAGTCGGCGTGGACTGTAGAAC ACTGCCAATGCCGGTCCCAAGCCCGGATAAAAGTGG AGGGTACAGTCCACGC
**Circ_gRNA(Scramble)**	GCCATCAGTCGCCGGTCCCAAGCCCGGATAAAATGG GAGGGGGCGGGAAACCGCCTAACCATGCCGACTGAT GGCAGAAAAAAAAAAGTGGGAGATTTATTGTCGGac agAGAGAAACTCTGCTGTGTCTGAAGGCAGATCTCCC

	TATAGTGACCCTGCCTTgcGACTGCCGGGACGAAATG<br>AGAGAAATCGTAACGGATGCGGAAGAGGCAGACAG<br>GTAAAAAAAAAACTGCCATCAGTCGGCGTGGACTGT<br>AGAACACTGCCAATGCCGGTCCCAAGCCCGGATAAA<br>AGTGGAGGGTACAGTCCACGC
**Circ_gRNA(AldoB148TGA)**	GCCATCAGTCGCCGGTCCCAAGCCCGGATAAAATGG<br>GAGGGGGCGGGAAACCGCCTAACCATGCCGACTGAT<br>GGCAGAAAAAAAAAAGTGCACATCGGCACGTGACCT<br>GCTTTCTTCTATGTGAGTAGTGTTCTTCCCAATGTGCT<br>ATACAAATAATTGAAGGCGCACGTGCAGTATAACTATA<br>AATAGTAATGCTGCCTTCCTCCTTCAGACAAAAAAAA<br>AAAAAACTGCCATCAGTCGGCGTGGATGTAGAACAC<br>TGCCAATGCCGGTCCCAAGCCCGGATAAAAGTGGAG<br>GGTACAGTCCACGC
**Circ_gRNA_ms2(mCherry190TAG)**	GCCATCAGTCGCCGGTCCCAAGCCCGGATAAAATGG<br>GAGGGGGCGGGAAACCGCCTAACCATGCCGACTGAT<br>GGCAGAAAAAAAAAAGTGGGAGATTCGGGCTCGGA<br>CAGAGAGAAACTCTGCTGTGTCTGACTTGGGATCTCC<br>CTATAGTGACCCTGCCTTCACGGGCCCGGGACGAAAT<br>GAGAGAAATCGTAACGGCTTGGCCTGAGGCAGACA<br>GGTAAAAAAAAAAACATGAGGATCACCCATGTacacac<br>acacCTGCCATCAGTCGGCGTGGACTGTAGAACACTG<br>CCAATGCCGGTCCCAAGCCCGGATAAAAGTGGAGGG<br>TACAGTCCACGC
**Circ_gRNA_ms2U(mCherry190TAG)**	GCCATCAGTCGCCGGTCCCAAGCCCGGATAAAATGG<br>GAGGGGGCGGGAAACCGCCTAACCATGCCGACTGAT<br>GGCAGAAAAAAAAAAGTGGGAGATTCGGGCTCGGA<br>CAGAGAGAAACTCTGCTGTGTCTGACTTGGGATCTCC<br>CTATAGTGACCCTGCCTTCACGGGCCCGGGACGAAAT<br>GAGAGAAATCGTAACGGCTTGGCCTGAGGCAGACA<br>GGTAAAAAAAAAAACATGAGGATCACCCATGTacacac<br>acacCTGCCATCAGTCGGCGTGGACTGTAGAACACTG<br>CCAATGCCGGTCCCAAGCCCGGATAAAAGTGGAGGG<br>TACAGTCCACGC
**Circ_ms2_gRNA(mCherry190TAG)**	GCCATCAGTCGCCGGTCCCAAGCCCGGATAAAATGG<br>GAGGGGGCGGGAAACCGCCTAACCATGCCGACTGAT<br>GGCAGacacacacacACATGAGGATCACCCATGTAAAA<br>AAAAAAGTGGGAGATTCGGGCTCGGACAGAGAGAA<br>ACTCTGCTGTGTCTGACTTGGGATCTCCCTATAGTGAC<br>CCTGCCTTCACGGGCCCGGGACGAAATGAGAGAAAT<br>CGTAACGGCTTGGCCTGAGGCAGACAGGTAAAAAA<br>AAAACTGCCATCAGTCGGCGTGGACTGTAGAACACT<br>GCCAATGCCGGTCCCAAGCCCGGATAAAAGTGGAGG<br>GTACAGTCCACGC
**Circ_ms2U_gRNA(mCherry190TAG)**	GCCATCAGTCGCCGGTCCCAAGCCCGGATAAAATGG<br>GAGGGGGCGGGAAACCGCCTAACCATGCCGACTGAT<br>GGCAGacacacacacACATGAGGATTACCCATGTAAAA<br>AAAAAAGTGGGAGATTCGGGCTCGGACAGAGAGAA<br>ACTCTGCTGTGTCTGACTTGGGATCTCCCTATAGTGAC

	CCTGCCTTCACGGGCCCGGGACGAAATGAGAGAAATCGTAACGGCTTGGCCTGAGGCAGACAGGTAAAAAAAAAACTGCCATCAGTCGGCGTGGACTGTAGAACACTGCCAATGCCGGTCCCAAGCCCGGATAAAAGTGGAGGGTACAGTCCACGC

Supplementary Table 4: Sequencing metrics, N50: The N50 value is the minimum read length required to cover 50 % of the reference sequence. Average base call quality score**=**The base call quality score is calculated using the Phred quality score formula $Q = -10\log_{10}(P)$ and is based on the average probability of incorrect calls (P) estimated by the Dorado basecaller's internal algorithms and models.

Sample	#reads (Mio)	# mapped reads (Mio)	N50	Average Base Call Quality
Oligo 1	1.5	0.38	140	13.4
Oligo 2	4.5	1.7	214	14.0
LCK-FUS$^{2–267}$-MCP-DKC1	20.5	0.98	1025	19.1
NES-DKC1	20.0	1.5	1130	19.4
Control	14.0	0.8	1032	18.6

Supplementary Table 5: RNA oligo sequences, (~U) denotes the modified base

**Oligo 1 (eGFP modified/mCherry unmodified)**	(GGG CGA UGC CAC C(~U)A GGG CAA GCU GAC CCU GAA GUU CAU C CUA CAA GGC CAA GUA GCC CGU GCA GCU GCC CGG CGC CUA C)$_n$
**Oligo 2 (eGFP unmodified/mCherry modified)**	(GGG CGA UGC CAC CUA GGG CAA GCU GAC CCU GAA GUU CAU C CUA CAA GGC CAA G(~U)A GCC CGU GCA GCU GCC CGG CGC CUA C)$_n$
