# Supplementary material for: Selective RNA pseudouridinylation in situ by circular gRNAs in designer organelles
Source: Nat Commun. 2024 Oct 24;15:9177. doi: 10.1038/s41467-024-53403-1 (PMC11502668; doi:10.1038/s41467-024-53403-1)
Supplement: Supplementary file 2 — Reporting Summary [file 41467_2024_53403_MOESM2_ESM.pdf]

nature portfolio

Corresponding author(s): Edward A. Lemke

Last updated by author(s): Oct 7, 2024

Reporting Summary

Nature Portfolio wishes to improve the reproducibility of the work that we publish. This form provides structure for consistency and transparency in reporting. For further information on Nature Portfolio policies, see our Editorial Policies and the Editorial Policy Checklist.

Statistics

For all statistical analyses, confirm that the following items are present in the figure legend, table legend, main text, or Methods section.

n/a	Confirmed	
☐	☒	The exact sample size (*n*) for each experimental group/condition, given as a discrete number and unit of measurement
☐	☒	A statement on whether measurements were taken from distinct samples or whether the same sample was measured repeatedly
☐	☒	The statistical test(s) used AND whether they are one- or two-sided<br>*Only common tests should be described solely by name; describe more complex techniques in the Methods section.*
☒	☐	A description of all covariates tested
☒	☐	A description of any assumptions or corrections, such as tests of normality and adjustment for multiple comparisons
☐	☒	A full description of the statistical parameters including central tendency (e.g. means) or other basic estimates (e.g. regression coefficient) AND variation (e.g. standard deviation) or associated estimates of uncertainty (e.g. confidence intervals)
☐	☒	For null hypothesis testing, the test statistic (e.g. *F*, *t*, *r*) with confidence intervals, effect sizes, degrees of freedom and *P* value noted<br>*Give P values as exact values whenever suitable.*
☒	☐	For Bayesian analysis, information on the choice of priors and Markov chain Monte Carlo settings
☒	☐	For hierarchical and complex designs, identification of the appropriate level for tests and full reporting of outcomes
☒	☐	Estimates of effect sizes (e.g. Cohen's *d*, Pearson's *r*), indicating how they were calculated

Our web collection on statistics for biologists contains articles on many of the points above.

Software and code

Policy information about availability of computer code

Data collection	FACS Diva version 9.0.1 - flow cytometry, dorado basecaller (v0.7.0) , minimap2 v2.28, samtools v1.16, NanoComp v1.23.125, R v4.2.3 , Rstudio v2022.7.2.576
Data analysis	FlowJo version 10.7.1 (BD Biosciences) - flow cytometry; Microsoft Excel 2016, GraphPad Prism 9.1.1 (225); R package pheatmap (version 1.0.12), R package ggplot2 v 3.4.4, Fiji v1.53.c, Python v3.10, trimmomatic v0.39, Bowtie2. v2.4.2, pysam package v0.22.1, custom code is provided as GitHub link https://github.com/AnWiercze/Pseudouridine_detection_Schartel_et_al_2024

For manuscripts utilizing custom algorithms or software that are central to the research but not yet described in published literature, software must be made available to editors and reviewers. We strongly encourage code deposition in a community repository (e.g. GitHub). See the Nature Portfolio guidelines for submitting code & software for further information.

Data

Policy information about availability of data

All manuscripts must include a data availability statement. This statement should provide the following information, where applicable:

- Accession codes, unique identifiers, or web links for publicly available datasets
- A description of any restrictions on data availability
- For clinical datasets or third party data, please ensure that the statement adheres to our policy

The data generated in this study are provided in the main text and Supplementary Information. Source data are provided with this paper. Sequencing data is available under Accession number PRJEB76145 in the European Nucleotide Archive (ENA) at EMBL-EBI.

Research involving human participants, their data, or biological material

Policy information about studies with human participants or human data. See also policy information about sex, gender (identity/presentation), and sexual orientation and race, ethnicity and racism.

Reporting on sex and gender	N/A
Reporting on race, ethnicity, or other socially relevant groupings	N/A
Population characteristics	N/A
Recruitment	N/A
Ethics oversight	N/A

Note that full information on the approval of the study protocol must also be provided in the manuscript.

Field-specific reporting

Please select the one below that is the best fit for your research. If you are not sure, read the appropriate sections before making your selection.

[x] Life sciences [ ] Behavioural & social sciences [ ] Ecological, evolutionary & environmental sciences

For a reference copy of the document with all sections, see nature.com/documents/nr-reporting-summary-flat.pdf

Life sciences study design

All studies must disclose on these points even when the disclosure is negative.

Sample size	Three independent biological replicates were used for flow cytometry experiments and western blots. 50000 live cells were measured in a single flow cytometry experiment. Direct RNA seq and BID-seq was performed once to confirm results.
Data exclusions	No data were excluded from the analyses.
Replication	Three independent biological replicates were used for flow cytometry and western blot experiments. All findings could be replicated
Randomization	not applicable
Blinding	not applicable

Reporting for specific materials, systems and methods

We require information from authors about some types of materials, experimental systems and methods used in many studies. Here, indicate whether each material, system or method listed is relevant to your study. If you are not sure if a list item applies to your research, read the appropriate section before selecting a response.

Materials & experimental systems

n/a	Involved in the study	
☐	☒	Antibodies
☐	☒	Eukaryotic cell lines
☒	☐	Palaeontology and archaeology
☒	☐	Animals and other organisms
☒	☐	Clinical data
☒	☐	Dual use research of concern
☒	☐	Plants

Methods

n/a	Involved in the study	
☒	☐	ChIP-seq
☐	☒	Flow cytometry
☒	☐	MRI-based neuroimaging

Antibodies

Antibodies used	donkey anti-mouse conjugated with Alexa FluorTM 488, A-31570 Thermo Fisher Scientific. 1:1000 dilution;<br>anti-myc, 1:1000, Cell Signaling Technology, 9B11<br>anti-mouse HRP (1:10000, Jackson ImmunoResearch, 715-035-150)<br>anti-FLAG (1:500, 9A3, CellSignaling)
Validation	Antibodies were validated by the manufacturers, validation data are provided on a corresponding manufacturer's webpage describing the antibody.

Eukaryotic cell lines

Policy information about cell lines and Sex and Gender in Research

Cell line source(s)	HEK293T (ATCC, CRL-3216)
Authentication	Authenticated by the manufacturer. Validation by morphology.
Mycoplasma contamination	All cell lines were regularly tested for Mycoplasma contamination, with negative results.
Commonly misidentified lines (See ICLAC register)	No misindentified lines were used.

Plants

Seed stocks	*Report on the source of all seed stocks or other plant material used. If applicable, state the seed stock centre and catalogue number. If plant specimens were collected from the field, describe the collection location, date and sampling procedures.*
Novel plant genotypes	*Describe the methods by which all novel plant genotypes were produced. This includes those generated by transgenic approaches, gene editing, chemical/radiation-based mutagenesis and hybridization. For transgenic lines, describe the transformation method, the number of independent lines analyzed and the generation upon which experiments were performed. For gene-edited lines, describe the editor used, the endogenous sequence targeted for editing, the targeting guide RNA sequence (if applicable) and how the editor was applied.*
Authentication	*Describe any authentication procedures for each seed stock used or novel genotype generated. Describe any experiments used to assess the effect of a mutation and, where applicable, how potential secondary effects (e.g. second site T-DNA insertions, mosiacism, off-target gene editing) were examined.*

Flow Cytometry

Plots

Confirm that:

☒ The axis labels state the marker and fluorochrome used (e.g. CD4-FITC).

☒ The axis scales are clearly visible. Include numbers along axes only for bottom left plot of group (a 'group' is an analysis of identical markers).

☒ All plots are contour plots with outliers or pseudocolor plots.

☒ A numerical value for number of cells or percentage (with statistics) is provided.

Methodology

Sample preparation	Samples were derived from HEK293T cells as detailed in the Methods section.
Instrument	LSRFortessa SORP, BD Biosciences

Software	DIVA Software version 9.0.1 for collection, FlowJo version 10.7.1 (BD Biosciences) for analysis.
Cell population abundance	50000 live cells
Gating strategy	First, the population of HEK293T cells was gated (using FSC-A x SSC-A parameters), and then a single cell population was selected (SSC-W x SSC-A). Next, live cells were picked (SSC-W x 405–450/50 channel). Then the analysis was dependent on the used reporter. details are supplied with the supplementary information

☒ Tick this box to confirm that a figure exemplifying the gating strategy is provided in the Supplementary Information.
